# Amlodipine-Induced Gingival Hyperplasia in a Young Male with Stage 5 Chronic Kidney Disease

**DOI:** 10.1155/2020/7801546

**Published:** 2020-02-12

**Authors:** Kevin John, Ajay Kumar Mishra, Karthik Gunasekaran, Ramya Iyyadurai

**Affiliations:** Department of Medicine, Christian Medical College, Vellore, India

## Abstract

Gingival hyperplasia is a rare finding in clinical practice. Nevertheless, when it occurs, it is a finding of great value as it can lead to definite clinical diagnosis. The present case is a 19-year-old male who was referred for further management of stage 5 chronic kidney disease. On evaluation, he was found to have gingival hyperplasia. He was evaluated for reversible causes of kidney disease, and since none were found, renal replacement therapy was advised. He had been taking amlodipine for blood pressure control. As this was presumed to be the cause of gingival hyperplasia, it was stopped and replaced by a combination of beta-blocker and prazosin. At six-month follow-up, he had complete resolution of gingival hyperplasia. Amlodipine as a cause of gingival hyperplasia is a rare occurrence. However, it is crucial to keep in mind such a possible side effect of this commonly prescribed antihypertensive drug.

## 1. Introduction

Gingival hyperplasia is a physical finding that is rarely encountered in clinical practice. However, once it is confirmed, it is an important sign as it can give a clue for the correct diagnosis of a patient. Over the years, various causes of gingival hyperplasia have been reported in the literature. The common causes of gingival hyperplasia are drugs, non-Hodgkin's lymphoma, acute monocytic leukemia, granulomatous diseases, and chronic periodontal infections [1]. This list can be further narrowed down with a focused history and physical examination. If a precise aetiology is established, it is often possible to avoid a biopsy, and treatment can be instituted by merely avoiding the etiological agent. In this context, it would be useful to know all the causes of gingival hyperplasia, both familiar and obscure. Here, we describe the rare case of a patient who presented with amlodipine-induced gingival hyperplasia in the background of stage 5 chronic kidney disease.

## 2. Case Presentation

A 19-year-old boy presented with nocturnal cough and bilateral lower limb swelling of 3 months duration. There were no complaints of frothy urine, decreased urine output, or hematuria. He was evaluated for these complaints at his hometown and diagnosed with hypertension and stage 5 chronic kidney disease. He did not have any addictions, allergies, or significant family history for kidney disease. He was started on 10 mg of amlodipine and referred to our hospital for further evaluation and management. After starting amlodipine, the patient noticed painless, gradually progressive, and irregular swelling of his gums with few episodes of bleeding while brushing his teeth. When he presented to us, he had been taking amlodipine for three months in total.

**Figure 1:**
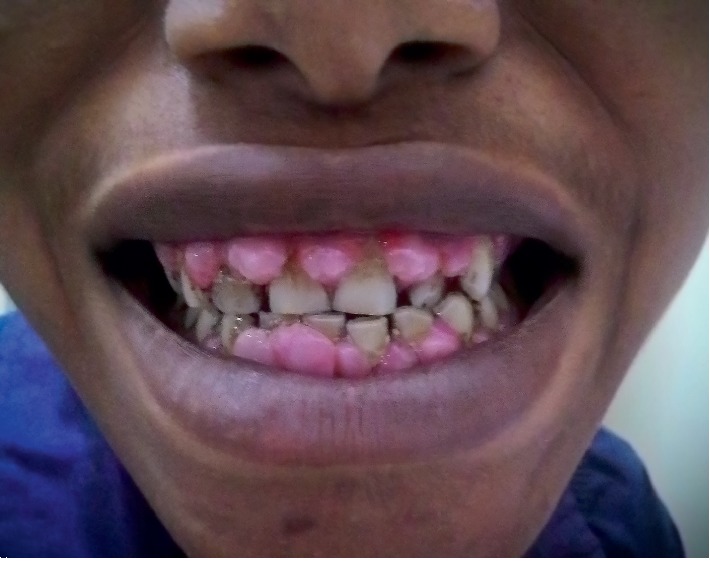
Photograph showing amlodipine-induced gingival hyperplasia.

On presentation to the outpatient department, he had a blood pressure of 180/100 mmHg in both upper limbs. His heart rate was 84 beats/min, respiratory rate was 18 breaths/min, and arterial oxygen saturation on room air was 98%. He was afebrile. He was noted to have pitting pedal oedema of both lower limbs up to the midcalf. On intraoral examination massive, painless, gingival enlargement involving both the arches, encroaching buccal, palatal, and lingual vestibular spaces was found (Figure 1). The gingiva was pale pink, firm in consistency and not tender on palpation. There were no other positive findings on examination of the other systems.

He was evaluated for treatable causes of chronic kidney disease. Causes of gingival hyperplasia that were considered included chronic periodontitis, drugs (calcium channel blockers such as amlodipine and nifedipine; cyclosporine and anticonvulsants such as phenytoin), acute monocytic leukemia, and non-Hodgkin's lymphoma.

The patient's initial blood investigations showed anaemia (Table 1). His creatinine was elevated, and an ultrasound scan showed bilateral shrunken kidneys with grade 3 renal parenchymal changes. The total duration of his kidney disease was not known. Kidney biopsy could not be performed given the small size and poor cortico-medullary differentiation. Hence, a complete evaluation of the cause of his kidney disease could not be conducted.

His blood leukocyte counts were normal, and this ruled out any blood dyscrasias. His blood picture did not show any blasts. He did not have lymphadenopathy. A diagnosis of stage 5 chronic kidney disease of unknown aetiology and secondary hypertension was made. A provisional diagnosis of amlodipine-induced gingival hyperplasia was made. Due to a clear temporal association, it was decided to assess response to the withdrawal of amlodipine before proceeding with further evaluation.

The patient was advised to take a low-salt renal diet. Amlodipine was stopped, and he was given a combination of atenolol and prazosin for blood pressure control. He was also started on febuxostat, torsemide, calcium and vitamin D supplements, phosphate binders, iron supplements, and erythropoietin. The need for renal replacement therapy was explained, and he was scheduled for the creation of an A-V(arteriovenous) fistula. The option of a renal transplant was also explained to him.

On follow-up after six months, the patient had complete resolution of gingival hyperplasia. A WHO-UMC causality assessment was performed, and the case fell into the “probable/likely” category. Proving “certain” causality would have required rechallenging the drug which would have been unethical. Therefore, it was considered to be sufficient evidence to attribute the cause of gingival hyperplasia to amlodipine. Chronic kidney disease was managed medically, and he was planned for an AV fistula creation for initiation of maintenance hemodialysis.

## 3. Discussion

Amlodipine is a dihydropyridine calcium channel blocker that is commonly prescribed as an antihypertensive drug. The common side effects of amlodipine include headache, dizziness, and palpitations. Gingival hyperplasia is a rare side effect of this drug. The prevalence of gingival hyperplasia caused by amlodipine had been estimated by Jorgensen in 1997 as 3.3% for patients in the United States of America, while a study conducted in India in 2014 by Tejnaniet al. arrived at a similar number of 3.4% [2, 3]. This suggests that the prevalence of amlodipine induced gingival hyperplasia is similar across populations of different race and geography, and has remained stable over time. Other causes of gingival hyperplasia are drugs (other calcium channel blockers such as nifedipine; cyclosporine and anticonvulsants such as phenytoin), non-Hodgkin's lymphoma, acute monocytic leukemia, granulomatous diseases (sarcoidosis and Crohn's diseases), granulomatosis with polyangiitis, chronic periodontal infections, pyogenic granuloma, benign neoplasms such as fibroma or papilloma, malignant neoplasms such as carcinoma or malignant melanoma, syndromic causes (Rutherford syndrome, Cross syndrome, Ramon syndrome, and Laband syndrome), pregnancy, and idiopathic gingival hyperplasia [4].

It was known that calcium channel blockers such as nifedipine could cause gingival hyperplasia. When amlodipine was introduced into the market, there were similar reports of gingival hyperplasia with this drug also. This was first reported in 1993 by Ellis et al. [5]. Since then, there have been various reports of similar cases. It is seen to be three times more common in males than in females [6]. There is a reported case of amlodipine causing hyperplasia of the hard palate as well [7].

The exact mechanism of amlodipine-induced gingival hyperplasia is not known. Various mechanisms have been postulated. These include defective collagenase activity due to reduced folic acid uptake, increase in adrenocorticotrophic hormone (ACTH) due to block in aldosterone synthesis, increase in keratinocyte growth factor, inflammation due to the concentration of drug in the crevices and bacterial plaques, as well as upregulation of transforming growth factor-beta [8–10]. Other factors such as dental hygiene, nutritional deficiencies, comorbidities such as renal failure (as seen with our patient) and male sex may also contribute to the gingival hyperplasia [6]. This could also be a biased observation as a large proportion of patients with chronic kidney disease are on amlodipine therapy as an antihypertensive.

Substituting the drug amlodipine with another antihypertensive remains the basis of management. In our patient, gingival hyperplasia had resolved entirely within six months of stopping the drug. Regression within three months of stopping the drug has also been reported [11]. This suggests that amlodipine-induced gingival hyperplasia is reversible with cessation of the drug alone. However, some advocate surgical management for a better aesthetic outcome. Gingivectomy or periodontal flap surgery can be performed. Maintenance of good oral hygiene and regular brushing of teeth are also of paramount importance. In summary, amlodipine is a potentially reversible cause of gingival hyperplasia, and this needs to be considered while evaluating patients who present with gingival overgrowth.

## Figures and Tables

**Table 1:** Lab investigations.

Investigations	Result (normal range)
Haemoglobin (g/L)	90 (140–175)
Total count (×10^9^/L)	6 (4.5–11.0)
Platelet count (×10^9^/L)	154 (150–350)
HIV, HBV, HCV serology	Negative
Serum creatinine (*μ*mol/L)	666.5 (62–106)
Erythrocyte sedimentation rate (ESR) (mm/hour)	22 (0–20)
Serum sodium (mmol/L)	138 (135–145)
Serum potassium (mmol/L)	5.2 (3.5–5)
Serum bicarbonate (mmol/L)	16 (22–29)
Serum uric acid (mg/dL)	5.8 (4–7)
Serum calcium (mg/dL)	7.81 (8.3–10.4)
Serum phosphorous (mg/dL)	5 (2.5–4.6)
Serum parathyroid hormone (pg/ml)	1305.3 (8–74)
c-ANCA(antineutrophilic cytoplasmic antibodies)	Negative
p-ANCA(antineutrophilic cytoplasmic antibodies)	Negative
Thyroid stimulating hormone (TSH) (*µ*IU/ml)	2.6
Serum total complement	Normal
24-hour urine protein (g)	3.3 (50–150 mg)
